# Non-Positive Experiences Encountered by Pupils During Participation in a Mindfulness-Informed School-Based Intervention

**DOI:** 10.1007/s12310-023-09591-0

**Published:** 2023-07-02

**Authors:** E.J. Miller, C. Crane, E. Medlicott, J. Robson, L. Taylor

**Affiliations:** 1 University of Auckland, Auckland, New Zealand; 2 University of Oxford, Oxford, UK; 3 OxfordHealth NHS Foundation Trust, Oxford, UK; 4 East London NHS Foundation Trust, London, UK

**Keywords:** Mindfulness, School-based interventions, Non-positive experiences, Harm, Adverse outcomes

## Abstract

Mindfulness-informed school-based mental health curricula show much promise in cultivating a positive school climate which supports the well-being and mental health of pupils and staff. However, non-positive pupil outcomes and experiences of school-based mental health interventions are often under-recognised and under-reported. This study sought to capture non-positive pupil experiences of a popular mindfulness-informed curriculum. Some pupils across all schools in the study described non-positive experiences, including having troubling thoughts and emotions, and not finding the programme effective. Contexts surrounding these experiences are explored and linked to existing literature, and subsequent recommendations for improvements are made, including the importance of having clear programme structure, definitions and aims, acknowledging and accommodating fidelity issues as best as possible, and better highlighting the potential for non-positive experiences and how they may be reduced.

## Introduction

### Background

School-based interventions (SBIs) are any programme, intervention or strategy applied in a school setting that are specifically designed to influence the emotional, behavioural or social functioning of students (Fazel et al., 2014). Schools are increasingly considered to be useful points of intervention for children and young people’s health due to their broad reach and potential cost effectiveness (Fazel et al., 2014). Mindfulness-informed SBIs are a group of programs based around the practice of mindfulness meditation, which was first manualised in the form of Mindfulness-Based Stress Reduction (MBSR) (Dunning et al., 2018; Kabat‐Zinn, 2003). Structural derivatives of MBSR, such as Mindfulness-Based Cognitive Therapy (MBCT), are known as Mindfulness-Based Interventions (MBIs) (Crane et al., 2016). The many ubiquitous mental health, school-based and app-based programs which often include elements of mindfulness practice, but are not structurally or fundamentally comparable to MBSR, are therefore categorised as mindfulness-informed programs.

While the trend towards the use of SBIs makes intuitive sense (Fazel et al., 2014), concerns exist around the large variations in efficacy and quality of different programs, including not knowing which programs work best for whom and why (Montero-Marin et al., 2022; Stallard et al., 2013). Importantly, non-positive pupil outcomes and experiences are under-recognised and under-reported in SBIs. One study looking at a universal cognitive behavioural therapy programme for reducing symptoms of depression in high-risk adolescents found that at 12 months post-intervention, there were greater symptoms of low mood in the ‘high-risk’ subgroup compared with the control group (Stallard et al., 2012). The authors observed that the intervention may have a potentially harmful effect when compared to usual school provision. Another meta-analysis of school-based social and emotional learning interventions did not look for non-positive experiences, while a further study of the UK Resilience Program had no exploration of non-positive experiences in the qualitative section of the study (Challen et al., 2011; Taylor et al., 2017). Non-positive experiences were not mentioned or addressed in several other overview articles of interventions (Fazel et al., 2014; Sanchez et al., 2018; Weare & Nind, 2011).

The rapid rise in popularity of MBIs, in both clinical and non-clinical populations, has been met with increasing concerns over their safety (Wong et al., 2018). Similarly, traditional meditation practices are being acknowledged as having non-positive experiences associated with their use (Wong et al., 2018). A recent study highlighted several challenging, difficult, distressing, and functionally impairing experiences of traditional Buddhist meditators (Lindahl et al., 2017). Non-positive experiences are often not monitored or reported in trials of MBIs, and there is often a focus on ‘positive effects’ of meditation in scientific research (Farias & Wikholm, 2016; Lindahl et al., 2017). Non-positive experiences of MBIs have been listed as increases in vulnerability; a sense that the difficulties experienced had increased in severity; a sense of failure; judging the practice as good or bad; finding the practices ‘too weird’; and misunderstanding of the basic cognitive aspects of the practice (Malpass et al., 2012). Children participating in meditation practices have also reported non-positive experiences. These include difficulty with wandering and fidgety minds (Cain, 2012; Coholic, 2011; Kempson, 2012; Tharaldsen, 2012); anxiety around revealing aspects of self to others (Cain, 2012), weird experiences (Cain, 2012); self-judgement and criticism (Dellbridge & Lubbe, 2009); difficulties with certain emotional responses (Kempson, 2012; Monshat et al., 2013); and findings that included participants not having clear expectations for the practices, not enough time given to participants to understand the practices, and finding that some practices were too rushed (Ruskin et al., 2017).

These qualitative accounts of non-positive experiences may also be reflected in some troubling results from recent randomised controlled trials (RCT’s) of a particular mindfulness-informed SBI called the ‘.b’ (pronounced “dot-be”) curriculum. More information about the. b curriculum is provided in the upcoming section. "the ‘.b’ Curriculum". While some early trials of .b reported encouraging results on psychometric testing (Kuyken et al., 2013), other more recent trials have showed nil effect or even negative effects compared to the control interventions. A 2016 randomised study of 115 participant pupils (mean age 13.66) who were taught .b found no outcome effects in the domains of anxiety, depression or eating disorder risk factors at three-month follow-up compared to the control arm, which consisted of normal pupil pastoral care or community projects (Johnson et al., 2016). However, self-rated anxiety was actually higher in the mindfulness group post-intervention across a range of subgroups. A second similar RCT conducted by the same authors also found no effects in primary outcome measures of anxiety, well-being, depression, weight/shape concerns, well-being and mindfulness at post-intervention, six- or twelve-month follow-up (Johnson et al., 2017). Between 2016 and 2018, a large and well-designed cluster randomised controlled trial called ‘MYRIAD’ (My Resilience in Adolescence), which recruited 85 secondary schools across the UK, and which measured data from over 7000 pupils aged 11–13, also found that the .b intervention was not only not more effective compared to the control intervention (teaching as usual) in improving mental health and well-being, but in fact it actually seemed to result in higher scores of hyperactivity/inattention (post-intervention and 1-year follow-up), higher panic disorders and obsessive–compulsive scores (post-intervention), lower levels of mindfulness skills (post-intervention), and higher teacher-reported emotional symptoms on the Strengths and Difficulties Questionnaire (SDQ) (at 1-year follow-up only) (Kuyken et al., 2022; Montero-Marin et al., 2022). It also found that young people who were identified as high-risk of mental health problems and were in the .b intervention arm of the trial seemed to report significant detrimental effects on risk of depression and well-being (post-intervention and at 1-year follow-up), which may also have been associated with increasing dose and reach (i.e. more exposure to the .b programme, such as by not skipping lessons, seemed to lead to worse outcomes). This lead the authors to conclude that .b should not only be not indicated as a universal intervention, but that it may actually be contraindicated for students with existing or emerging mental health symptoms.

Authors have accordingly argued for more conceptual clarity around harms in MBIs, and similarly that further clarity is required to better understand how and why universal, mindfulness-informed SBIs may exert their effects, including who may benefit most, and why (or why not) (Baer et al., 2019; Tudor et al., 2022). This includes calls to examine for potential moderators (e.g. the wider school context and characteristics of the schools, teachers, and students), implementation factors (e.g. intervention fidelity, dose, quality, reach and mindfulness practice) and mediators (e.g. mindfulness skills and executive function) (Tudor et al., 2022). There is subsequently an urgent need for a deeper understanding of non-positive experiences of young people participating in MBIs in their schools in a way that particularly foregrounds the voices of young people and enables them to describe their non-positive experiences in detail.

### The ‘.b’ Curriculum

The .b curriculum is a mindfulness-informed curriculum designed for 11–18 years olds which is run by a not-for-profit UK organisation called the Mindfulness in Schools Project (MiSP)(MiSP, 2022). It was chosen for this research because it is the mindfulness curriculum with the most widespread use within the UK, and alongside other similar courses run by MiSP, has been taught to over 5000 teachers and at least 350,000 pupils both in the UK and across the globe, with the aim of targeting one million pupils in total, and having already been translated into twelve languages (MiSP, 2023). It is also the curriculum studied in several large RCT’s, as discussed above. It consists of 10 lessons, each one ranging from between forty minutes to one hour, and is taught to all pupils (i.e. pupils are not screened or indicated beforehand) weekly during school hours by a regular school teacher who has been trained in the .b curriculum. Pupils are also encouraged to complete home practices supported by online video teachings. Lessons typically follow a format of introducing a new topic or idea (such as the skill of being able choose where to direct one’s attention) which is then further explained through either teacher led discussion (based on scripted notes in the teacher’s handbook) or an animation, which incorporates the use of quotes, poems, anecdotes or movie clips. Pupils are then instructed to perform either a practice or fill in a worksheet. Experiences and ideas are then shared with the class and the teacher (MiSP, 2022).

### Study Aims

The primary aim of this study was to explore how young people described any non-positive experiences they may have encountered during participation in a standardised mindfulness-informed SBI named .b. The secondary aims were to understand the relationship between the way young people described and conceptualised their non-positive experiences and the broader context of the SBI.

## Methods

### Design and Analysis

This project was designed from a qualitative perspective, using an ethnographically-informed case study approach (Yin, 1994). The case for this project was identified as four secondary school classes participating in the study, and the unit of analysis—that is, the major entity of enquiry within the study—was the interaction between the teacher, the curriculum and the pupil. These classes were chosen because they represented the typical school classroom where the .b programme was being implemented. Since the aim of this study was primarily to capture experiences as they occurred within the social context of the typical school classroom, an ethnographic approach was felt the most appropriate approach to inform data collection—consistent with ethnography as a ‘cultural description’ (Wolcott, 2008). While the study does not constitute a typical full ethnography, aspects of the design, such as the embedding of the researcher within the classroom to better understand the whole context, were influenced by this approach to data collection. The data set was coded using interpretive phenomenological analysis (IPA) (Smith et al., 2009).

Anonymised interview recordings were transcribed by an independent third-party company. These transcripts, alongside classroom observation notes and pupil diaries, comprised the data set for each school. The lead researcher(E.J.M.) immersed himself in the data and undertook initial coding of the printed data set. The data set for each school was initially coded by hand, with codes being written down the left hand side margin of the interview transcripts, observation notes, and diaries. This process was then repeated. Initial themes relating to the data set were then generated on a separate piece of paper by hand. These initial themes were then transferred to Microsoft Word (outline mode) and refined to produce a second set of themes. Once each school’s data set was outlined on Microsoft Word, these data sets were combined to produce four final meta themes relevant to all three schools. This produced one overall picture of the entire data set, which was generated between Feb 2018 and June 2018, and did not aim to compare time points within schools or between schools, or describe individual pupil case studies.

Throughout this process, multiple group coding meetings were held in order to discuss validity of emerging themes and inter-rater-reliability of codes. This included one group coding meeting in April 2018 where all five researchers discussed their own individually generated hand written codes and themes for the interviews from School 1 and further meetings in May and June 2018 with E.J.M., C.C. and L.T. discussing emerging codes and themes from the other two schools. During this process, the similarities, differences and subjective validity of codes were discussed and reasoned through, resulting in, for example, some codes or themes being omitted, or others being combined or created into new categories based on the iterative process. There were no critical disagreements or differences throughout this process that required mediation.

An audit trail was maintained during the entire process of this study, and a bracketing interview was also conducted by the researcher and a supervisor, which included challenging the lead author on their own experiences with mindfulness meditation, whether they had encountered any non-positive experiences themselves, and why they wanted to do a project such as this. The lead researcher at the time was a medical doctor who also did clinical work in the field of psychiatry, who also had an established personal practice of mindfulness meditation and an interest in researching and developing school-based mindfulness interventions. He had no personal experience of non-positive outcomes of mindfulness meditation. Authors E.M., C.C. and L.T. were researchers who also worked on a large trial of mindfulness interventions in schools, and author J.R. was an additional but separate researcher with a background of qualitative research in education who provided oversight and support to the present study.

### Methods

Each session of the .b programme in each school was observed and notated in a semi-structured format by the lead researcher. The researcher sat at the back of the classroom behind all the pupils and transcribed notes in a semi-structured framework, incorporating comments on general environment, appearances, verbal behaviour, physical behaviour and interactions. The researcher’s reflective commentary was recorded after each session in the classroom observation notes as well as in a personal research diary. Each session was also video-recorded and analysed to assist with providing a more detailed assessment of quality of teaching in general (as coded by the Mindfulness-Based Interventions Teaching Assessment Criteria Teacher Addendum (MBI-TAC-TEACH).

Before each session (excluding the first session) and after each session (excluding the last session) all pupils were asked to fill in a journal. The journal before each session asked the pupils three short questions about their experiences during the week since the previous session, including related to any home practice they may have completed. The journal after the session asked three similar questions related to the pupils’ experiences during that session (Table 1).

**Table 1:** Outline of the 10 .b lessons (adapted from Kempson, 2012)

Lesson	Key learning points	Key practices
Lesson one—an introduction to mindfulness	Brief theoretical background to mindfulness and its potential applications Brief outline of the lesson contents	N/A
Lesson two—playing attention	Directing attention (metaphor of spotlight is used) Exploring and investigating bodily sensations and the breath Aiming and sustaining attention through firm, patient kind repetition	Simple directing of attention—this exercise requires pupils to pay attention to discrete parts of the body and to explore the sensations they may experience) Aiming and sustaining attention for 2 min—this exercise requires pupils to direct their attention to a particular sensation (e.g. breath) and subsequently to redirect it when attention wanders Counting breathes in 1 min—this exercise requires pupils to count the number of breaths they take in one minute
Lesson three—taming the animal mind	Explore idea that the mind has a life of its own that is outside our control Curiosity, kindness, openness and acceptance can help us deal with fluctuating mind states It is possible to anchor attention in lower half of body to increase calm when the mind is stormy	FOFBOC (feet on floor, bum on chair)—pupils are guided to pay initial attention to their feet and lower limbs, and then gradually expand their awareness to other sensations throughout their body
Lesson four—recognising worry	Recognising the impact of cognitive distortions (e.g. interpreting and storytelling, rumination and catastrophising)	7–11—this exercise requires pupils to pay attention to their breath while they inhale for 7 s and exhale for 11 Lying down body scan (Beditation) – in this exercise pupils are lie on their backs with their arms by their sides. They are then guided through a meditative exercise, paying attention to various bodily sensations and to observe their mental reactions
Lesson five—being here now	Understanding the impact of habitual and automatic modes of functioning (metaphor of autopilot is used) Developing appreciation of present moment experiences (savouring)	Mindful Mouthful—during this exercise, pupils are required to taste different items of food (firstly a Malteser, secondly a piece of chilli or onion and lastly a raisin). Pupils are encouraged and guided to explore, in as much detail, their physical and metal experience, both in anticipation to tasting and while eating ‘.b’ exercise—this exercise requires pupils to walk around, pretending they are stressed and busy. When the teacher blows a whistle they are to stop, pay attention to their feet on the ground, feel the sensations of their breathing and pay attention to how they are presently feeling
Lesson six—moving mindfully	Exploring the application of mindfulness to daily activities Investigating the effects of slowing down and paying conscious awareness to our actions	Samurai walking—this exercise requires pupils to walk 10–20 m focusing on the physical sensations in their legs and feet Routine revisited—as a purely home-based task, pupils are requested to choose one activity they do regularly and to purposely do it more slowly, paying attention to their sensations and reactions
Lesson seven—stepping back	Recognising our stream of consciousness and re-perceiving the nature of our thoughts Recognising habitual thought processes Depersonalising thought/ stepping back	Listening to thoughts as sounds—pupils are encouraged to observe their stream of consciousness and attempt to perceive thoughts as impersonal sound Top ten tunes—pupils are encouraged to notice reoccurring thoughts and list the 10 most prevalent Seeing thoughts as clouds—pupils are encouraged to depersonalise thoughts and see them as passing clouds, letting them come and go freely without reacting to them
Lesson eight—befriending the difficult	Understanding stress and how it impacts an individual Learning to accept and 'be with' difficult emotions Responding rather than reacting to emotions	Identifying stress signature—pupils are directed to imagine an imminent stressful experience and asked to notice and record how stress manifested in their bodily sensations and their breath
Lesson nine—taking in the good	To encourage an appreciation of good things in life Even ordinary things can be ‘good’ if we pay attention To look at the advice of people who have managed to appreciate the good even in awful circumstances Practice ‘taking in the good’ so you can experience it directly	Mindful eating—grape—including an appreciation of all the conditions that led to the grape being present in their hand. Notice experience of eating a grape and mind and body when doing so Gratitude practice—bring to mind a person, pet or place that you love. Notice sensations in body and breathe with them. Look for feelings of calm and notice sensations of wellness. Make a deliberate decision to attend to the good. Say thank you Notice any bodily responses to feelings of appreciation, gratitude or joy Optional—write gratitude text/letter/call
Lesson ten—pulling it all together	Identify what has been most useful about the course Consider areas of life to apply mindfulness skills Provide feedback	FOFBOC Mindful reflection on questions: • What have you learnt that has been useful? • Could mindfulness practice help in particular areas of life? • What can you appreciate? • Where could you benefit from responding rather than reacting? • What would you like to remember for the future? Habit breaker mindful eating of chocolate

Focus groups and interviews were conducted in a semi-structured format between mindfulness lessons five and six and after the 10^th^ (final) lesson. Focus groups were conducted with groups of five pupils (the classroom teachers selected the combinations of pupils present in each focus group, which was based on selecting pupils who were more likely to have appropriate behavioural interactions within each group while participating) with two or three interviewers (between E.J.M, E.M., C.C. and L.T.), all of whom participated in asking questions. Following the second focus group interview, pupils were immediately invited to participate in an individual interview with an interviewer, to explore individual experience and meaning making more deeply. One-to-one discussion was facilitated by referring to pupils’ previous responses in focus groups as well as proceeding diary entries.

### Recruitment and Participants

A large pool of schools, who were known to be actively implementing the .b mindfulness programme, were contacted and invited to participate in the study. Twenty .b mindfulness teachers in the region were also emailed directly asking if they would participate. Three schools that were delivering the programme in the relevant academic terms to permit data collection were recruited. These schools were selected from a broader group of schools and teachers expressing initial interest to ensure a spread of school funding type, pupil age, socioeconomic status and previous exposure to mindfulness and .b.

Four classes in three schools were ultimately recruited. The data and school characteristics of these are summarised in Table 2. Class 1 was a year 7 class in a secondary school where pupils were taking part in the .b programme as part of their normal school provision (i.e. a compulsory component of the school curriculum). The programme was delivered by a familiar member of staff who normally taught humanities and social sciences (designated Teacher 1 in the results). Two pupils who did not consent to the study sat in an area of the class behind the researcher and the video-recording. Class 2 was a year 11 group in a secondary school where pupils who were identified by the school as having some low-grade issues such as stress around exams were invited to participate in a .b programme voluntarily. Four pupils participated in the first focus group interview and five in the second round and individual interviews. Two teachers co-delivered the programme, sometimes swapping week to week, other times swapping within a lesson, to deliver the .b programme, in the pupils’ ‘tutor’ time of 40 min (these are designated Teacher 2a and 2b in results). Classes 3 and 4 were separate year 9 classes both in an independent secondary school with the highest quality rating from the Independent Schools Inspectorate. It was compulsory for these pupils to participate in the .b programme. The .b programme for each class was delivered by the same teacher, who also taught other lessons in the school (designated Teacher 3 in results). In summary, the study represented pupils between the ages of 11 and 15, in both single and mixed gender classrooms, and representing a range of socioeconomic deciles. Similarly, the context of the pupils’ participation in the programme, class size and usual teacher role also differed.

**Table 2:** Summary of school characteristics

	Number of participants	Pupil demographics	School characteristics	Quality of teaching rating
School 1	24 (diaries & interview) 9 (diaries and observation only) 2 (diaries only) 14 pupils participated in the first round of focus group interviews (due to one pupil being absent) and all 15 participated in the second focus group and individual interviews	11 males and 13 females (self-identified) Age 11–12	Ofsted rating ‘Good’ 27.5% free school meals 1% first language not English Index of Multiple Deprivation rank of 12,691	‘Competent’
School 2	7 (diaries and interview) Four pupils participated in the first focus group interview and five in the second focus group and individual interviews	6 female and 1 male (self-identified) Age 15	Ofsted rating ‘Good’ 7.8% free school meals 12% first language not English Index of Multiple Deprivation rank of 13,906	‘Competent’
School 3	Class 3: 15 pupils, all consented to diaries, observations and interviews. 12 pupils were present and participated on the day of the focus group interviews Class 4: 19 consented to observations, video recording and diaries and 15 consented to focus group interviews, of which 14 presented and participated on the day	All male aged 14 (self-identified)	Ofsted rating ‘Outstanding’ Index of Multiple Deprivation Rank of 20,568 First language not English data unavailable 0% free school meals	‘Competent’

#### Key

Ofsted—Office for Standards in Education, Children’s Services and Skills.

Index of Multiple Deprivation rank recorded in 2015 (where 1 is the area/neighbourhood in the UK with the highest level of deprivation, out of 32,884 areas. This is a relative measure of deprivation (incorporating weighted measures of data such as employment and crime rates), meaning it can tell if one area is more deprived than another, but not by how much—i.e. a small area with a rank of 250 is not half as deprived as an area with a rank of 500). (Communities & Government, 2015).

Free school meals UK national average of approximately 17. (Communities & Government, 2015).

Competency rating as per MBI-TAC-TEACH.

### Data Collection

The researcher was able to observe all lessons in School 2 and all in School 1 except for lesson 3, due to a last-minute timetabling change. The two classes in School 3 only had lessons 2, 4, 6, 8, 9 and 10 observed, due to timetabling clashes.

School 1 and 2 followed the full interview protocol described in the methods, while School 3 had only one set of focus group interviews during week 9 (for class 3) and week 10 (for class 4). There was no middle set of interviews and no individual end interviews due to timetabling conflicts and time availability of pupils. A modified focus group proforma was subsequently constructed for use in School 3, which was a combination of the existing first and second round interview proformas.

Data were collected between November 2017 and June 2018. Information and consent forms were generated for school headteachers, classroom teachers, and parents, while an information and assent form were generated for pupils. These were emailed to the schools followed by collection of hard copies with signatures. For one school, the parental and pupil information consent and assent forms were generated using the Qualtrics online software, and an ethics amendment was sought and approved for this. Safeguarding and risk matrix assessments were completed to allow for awareness of and proper management of any non-positive experiences that may require further attention—these were to be referred to the classroom teacher or safeguarding officer. The SD card used to video-record the pupils was stored inside a lock box that was kept with the classroom teacher. Other information such as observation notes used by the researcher were transported securely and stored in a locked drawer at the research facility.

## Findings

Through the coding process listed above, four themes were identified. These were non-positive experiences, pupil contexts, school contexts and the curriculum. The list of themes are displayed in Table 3. Key examples only are included in the text with additional examples included in the "Appendix". Contrasts and variations of pupils’ experiences between different schools, as well as any changes experienced throughout the course, are also discussed.

**Table 3:** Themes

Themes	
*Non-positive experiences* Some pupils had trouble with certain experiences relating to their thoughts, feelings and sensations; e.g. ‘I found it difficult not to cry’	
*Pupil contexts* Pupils’ interpretation and understanding of the course, as well as other factors that may have impacted their experience	
*School contexts* Factors relating to teaching the course may have impacted on pupil experience	
*The curriculum* Factors relating to the content and structure of the curriculum may have impacted on pupil experience	

Key for quotation references:

FG, focus group (either FG1 or 2 for School 1 and 2, or just FG for School 3 as only one time point); DE, diary entry; II, individual interview; TI, teacher interview; ON, classroom observation notes

### Non-Positive Experiences

A number of non-positive experiences were described by pupils. These are described and explored below, taking into account the contexts in which pupils were operating and the similarities and differences between pupil experiences.

A salient theme emerging in some pupil accounts was the description of an increased prevalence, awareness of, or triggering, of thoughts described as ‘negative’, ‘bad’ or in one case, ‘evil’. For example, one pupil described difficulties with a practice that was designed to illustrate mindfulness of thoughts. In this practice, a metaphor is introduced in which thoughts are described as like buses passing by, which one can choose to engage with (‘get on the bus’) or simply observe from a decentred perspective (‘watch the bus go by’). This pupil described that:

[The] thought bus one kind of made me really worried, because I started to notice when the bad thoughts came, and like they stay in my mind all day and I couldn’t get rid of them… when Miss said ‘Try and notice the good thoughts’ I found it really hard to like not think about the bad thoughts, because that would just stay in my brain…. [FG 2, School 1, Pupil 11, female]

Another pupil had a similar experience, seeming to describe that even the mentioning of ‘bad thoughts’ by the teacher triggered a fixation on bad things:

When we did the thought buses, when Miss was like ‘Think about the bad thoughts, and think about the good’, I couldn’t really focus on the good things. [When asked whether this had changed over time, she said]… I think I’m improving a lot, slowly, but I’m doing it. I think sometimes I’m just quite negative. But you’ve got to be positive about life and that’s the thing. [II, School 1, Pupil 18, female]

Another pupil, again describing a cycle in which attempted suppression or blocking out of thoughts led to them rebounding, said:

If I have to think about it, it makes me think more about that thing, then I worry about it….. I think it’s the thought buses…. ‘cause if I try to block something out, I think about it even more. [II, School 1, Pupil 7, female]

Another example came from a pupil [JE, week 3, School 1, Pupil 24, male] who reported in his journal that he was ‘blocking out evil thoughts’ and ‘ignoring bad thoughts’. In response to the diary question ‘Did you have any experiences this .b lesson that were difficult or challenging?’ he wrote ‘to stop being nervous or scared about my thoughts.’

When interviewed this pupil perceived there to have been beneficial effects of the programme, but described this in a rather mixed way, as he also reported a time when it made a difficult thoughts worse:

There was one thing that got me quite upset, but now I’ll think about it, I’ll notice that it’s a bit emotional, but I’ve got to look on the bright side of things and notice the good instead of the bad. There was [also] a time where it didn't really help. 'Cause I was trying. But it just made it even worse. [II, School 1, Pupil 24, male]

Furthermore, a number of pupils made statements or comments which suggested that they felt judged in some way by the programme. For example Pupil 8 from School 1 described the programme by saying:

It’s practically saying ‘You’re a grownup now, grow up, hurry up, get up’, it’s being mindful, ‘Don’t be a child.’ [II, School 1, Pupil 8, female]

This pupil explained that she was encountering a difficult grieving situation at home relating to the recent death of her grandmother (Nan):

I know all the tasks and that are to help us with our emotions, all the breathing. It helped me with other emotions, not happy ones, like sad ones, about my Nan. I’ve been crying a lot more at home, because I’m thinking about a lot more stuff, because my Nan… I don’t really think about it unless I’m doing .b, because with .b it says to think about your emotions, think about your breathing, but when I do the .b at home it’s just like I think about my Nan, and she died in a really bad way. [II, School 1, Pupil 8, female]

Another pupil [School 2, Pupil 3, female], who stated regarding her present mental health that ‘I just have angry and that’s all I have. I don’t know’, and later that ‘When I was younger, I would get excited about things. Now, I just stay on a constant nothing’, described a sense that the course was not validating her actual present emotional experience:

I wouldn’t use it because, I don’t know, when I’m angry, I don’t want to be like ‘Oh, let me calm down,’ I’m like ‘Nah, I’m good at being angry’. [FG2, School 2, Pupil 3, female]

This was then reinforced by a peer in the focus group at this school:

I think sometimes it’s nice to feel the emotion – as in you’re actually there. Depending on what’s happened, sometimes I feel like it’s okay to actually be angry about something. [FG2, School 2, Pupil 6, female]

In both cases, echoing the findings concerning difficult thoughts, to some extent this appears to relate to the pupils’ interpretation of the intentions of the course as being intended to reduce negative thoughts and feelings (through blocking or ignoring) and hence calm them down. There may have been an element of implicit behavioural control from the school also present here. This pupil’s sense that she should ‘calm down’ may have ultimately translated across into the perceived lack of effectiveness from the course—that ‘nothing helps’.

A number of pupils also found the course or particular practices to be ineffective or to be at least ambivalent, which are summarised in Table 4:

**Table 4:** Summary of quotes of mixed effectiveness

Pupil	Quote
FG, School 3, Pupil 4, male	I’ve tried it and I don’t really feel like it’s working for me
FG, School 3, Pupil 5, male	I only really did the home practice when we got set a task, and even then I didn’t do all of it because I didn’t feel that much of an impact happening
DE, School 1, Pupil 15, weeks 1, 4, 6, 7	None of them are working for me - no practices ever work for me to be honest

The lack of effectiveness also seemed to translate into a lack of a sense of personal growth or change after doing the course. Take the following examples from pupils from School 3 all taken after the course finished:

Pupil 7: I don’t really get many experiences in there. It’s just been normal... I don’t really feel anything. I just go through the motions I guess.

Pupil 8: I don’t really use any of the things (practices). I wouldn’t say I’ve changed at all since we started it.

Pupil 9: I don’t think I’ve changed either. It’s not a life changing thing being able to drop your focus into your feet. So it’s kind of like, ‘I can do this’ but it hasn’t changed my life. [FG, School 3, pupils 7, 8 and 9, all male]

### Pupil Context

As explored above, non-positive experiences often were accompanied by a particular interpretation of the course content. These interpretations, including how they may relate to the actual content delivered, are explored in more detail in this section.

Firstly, some pupils had heard of mindfulness or meditation before, and had preconceived ideas about what it was or was not. This in turn may have influenced their interpretation of some of the lessons. For example, some pupils had learnt a different mindfulness curriculum at previous schools—Pupil 18 from School 1 was able to articulate this:

I thought it was a bit confusing, because I thought mindfulness was different, because I did it at primary and I didn’t know what .b actually meant, so I thought it was a bit confusing, because I didn’t know that you could do a .b mindfulness. [Before] We had to picture a beach, and in the lesson we do now we can do practicals, like breathing and things like that. So it’s quite different. [FG1, School 1, Pupil 18, female]

Pupils also described not really knowing why they were doing the .b course. Pupil 14 from School 1 explained:

So the whole thing at first, doing the meditating instead of doing our regular class, ‘Why are we doing this?’ It just all seemed weird and uncomfortable to do at the time… I think if we were told a little bit more about it before we started, I think it would have been all okay to do it. [FG 2, School 1, Pupil 14, male]

A similar example from pupils in School 3:

Pupil 13: I don’t think he said anything about why we’re doing it. He just said we’re doing this…

Pupil 8: Sort of made us, forced us. [FG, School 3, Pupil 13 and 8, both male]

This unclear sense of purpose for some pupils, perhaps coupled with preconceived ideas, may have contributed to how pupils interpreted some of the teaching points of the course, and impacted on their experience.

Subtle ways in which pupils could vary in their interpretation of course content can be illustrated by the following examples:

The first example comes from an exchange between the teacher and pupils of School 3, where the teacher was attempting to make the point that shifting attention away from rumination can sometimes help one to become ‘unstuck’ from thinking and bring a sense of psychological change or growth. The teacher told a story about a boy he had previously taught, who at the time was suffering from negative ruminating thoughts, as a close family member was dying. The teacher explained how he told the boy to ‘just notice (the thoughts) next time’ rather than ‘getting so caught up in it’, to which, sometime later, the boy returned to the teacher and told him that the practice had really helped him. However, later in the lesson, when the teacher asked the pupils what they could do about an email that they are worried about, a pupil, in rather perturbed fashion, replied ‘Delete the email’. In the second class at School 3, the teacher repeated the story and asked the same question, to which another pupil almost identically sardonically answered ‘Do something about it’. Later, that same pupil wrote in their diary:

Mindfulness seems to be about ‘dealing with negative emotions’. However, I think a more effective approach would be to actually deal with the problem, instead of ‘meditating’ in a dark room. Mindfulness may help some people ‘deal with their emotions’ but in my opinion it is ineffective – it being a placebo effect that may relieve ‘symptoms’ of a problem, instead of actually solving the problem, which would allow the symptoms to disappear completely. [DE, School 3, Pupil 7, week 2]

He also wrote in his diary at a later stage of the course:

If you don’t think about your problems, how are you going to solve them? [DE, School 3, Pupil 7, week 4]

This example nicely illustrated how at least two pupils in this session interpreted the meaning of the story as to block out or ignore a bad or difficult thought, even though the pupil in the story clearly benefited from shifting his attention away from the worry. The pupil’s reflection, that shifting attention may not actually help solve the underlying problem, was similarly expressed by Pupil 8 from School 1.

A similar scenario emerged from School 2 in the following example, taken from in-class observation notes, regarding a pupil (Pupil 7) who later dropped out of the course:

During a class discussion, Pupil 7 asked the following question after doing a practice: ‘I don’t understand why your mind is not supposed to think about certain things – like it is good to think about certain things that are happening in your life’. The teacher quickly answered the question, as it was the end of the lesson – ‘It gets better with practice’. [ON, School 2, week 3]

This is also an example of how school context, such as a teacher not having enough time to explore the phenomena in detail, can impact on pupil experience. Similar to the pupil in School 3, this pupil also perceived the practice as ‘not thinking about certain things’. The teacher wasn’t able to explore meaning and context with this pupil further, perhaps due to time as it was the end of the lesson. As stated earlier, this pupil left the course before interviews occurred so her understanding of this could not be explored further with her during interviews. School context is also explored in more detail in section "school contexts"

As alluded to earlier, pupils from School 1 seemed to have interpreted some instructions, particularly the thought bus exercise, as having a primary goal of not getting carried away on bad thoughts, and instead to focus on positive thoughts. The following passage illustrates Pupil 8 explaining how she disagreed with this logic:

Isn’t it like all of your thoughts are carrying you away somehow, even a good thought? Say you’re having a really good thought, like we won the football match, it could take you onto a bad thought as well, like we could have got loads more goals if we’d tried better, we could have got less injuries if we’d tried better and stuff like that. So it’s like all thought buses, whether you go on the good or the bad, the bad can turn into the good and the good can turn into the bad, so all of your thoughts would need to stop if you’re going to do like what Miss says, you’d need to stop thinking altogether, because everything can be dangerous, no matter what you think. [II, School 1, Pupil 8, female]

Here, Pupil 8 insightfully describes that ‘what Miss says’—to stop thinking bad thoughts and instead to think good thoughts—would be impossible, because even good thoughts can turn into bad thoughts. Hence, she concluded that one would have to stop thinking completely in order to not think anything bad.

She also critiqued the intention or desired outcome of the thought bus exercise of lesson 7, which she interpreted as blocking or not thinking about bad thoughts:

No matter what it’s always going to be there in your head, and if you don’t think about it now, what’s going to stop it coming up more seriously in the future? [II, School 1, Pupil 8, female]

Pupil 8 was the pupil who earlier described an experience of sadness about her grandmother’s death when doing .b at home. The fact that she didn’t raise this in class, or that it wasn’t mentioned as a possible effect, is an important point worth consideration. Like other pupils, she seemed to struggle with these difficulties in isolation. Her possible sense of being judged and invalidated potentially stemmed from how she may have interpreted the nature of some lessons—to block or ignore certain thoughts or feelings, or indeed to focus only on ‘positives’—which is what she potentially meant when she earlier described the school asking her to ‘grow up’ and ‘not be a child’.

Pupil 8 seemed to realise this herself, when she gave a brief critique of the curriculum:

No matter what it’s [the difficult thoughts] always going to be there in your head, and if you don’t think about it now, what’s going to stop it coming up more seriously in the future? Say I block a thought about the dreams, what happens if they get worse and worse and I keep on blocking them, and then when I try to get a job I turn paranoid, like I keep on seeing it because I haven’t been able to talk about it. That would ruin my life, because if I don’t sort it out now then it may lead to something way worse in the future. [II, School 1, Pupil 8, female]

This is an example of a very similar interpretation to the first two examples pupils from School 2 and 3 above.

Some pupils also described not understanding certain teaching points, while others described not finding the teaching points relevant or useful to them. Take the following example from Pupil 14 in School 3:

I think a couple of the animations that we watched, because you’d watch them and then our teacher would tell us, would ask us that they mean and what’s happening in them, and often at times I wouldn’t know what, I get the general idea but then I wouldn’t get the actual point that was being made. [FG, School 3, Pupil 14, male]

Pupil 14 described not understanding the point that was being made, but he didn’t ask for clarification or further understanding. This may have impacted on his interpretation and experience of the course.

### School Contexts

Since schools were a medium between the pupils and the content of the curriculum, school-related factors which may have contributed to pupils’ experiences of non-positive experiences, such as the delivery by teachers, are explored here.

The intention schools had for delivering the .b programme to their pupils differed to some degree from what the pupils believed it to be. In general, while many pupils either didn’t know or vaguely sensed it had something to do with calming, relaxation or ‘reducing stress’, the teachers generally had the perspective that it was about addressing some mental health problems in their pupils. However, the exact similarities and differences in the understanding of these terms between teachers and pupils was lacking between the two parties. Both teachers from School 1 and School 3 were unsure if pupils understood why they were doing the course, as this example from School 1’s teacher demonstrates:

I don’t think they probably realise. I reckon we need to do an audit on it to see what they do. I don’t think we’ve ever asked them, what is their awareness of it and things. [TI, School 1]

Teacher 1 shared that the school was concerned about the rate of self-harming, cutting, and general levels of anger in the pupil group, and that the programme was brought in to help address this, particularly while waiting for other service referrals:

Getting more instant help and instant response than we can do with the students while they are waiting for their CAMHS [child and adolescent mental health service] referrals. [TI, School 1]

She went on to describe .b as ‘mental health first aid’ and that teachers in general were not able to cope fully with the mental health problems they were presented with. This was reinforced by Teacher 2a from School 2, who acknowledged the pressure they felt in order to help with these problems:

External agencies are massively overwhelmed… I think as a country we have completely underestimated the size of this problem of mental health in young people. I think that mindfulness, while it’s still important to work in conjunction with those things, it’s one to one support that they need, because those agencies are so overwhelmed. [TI, School 2]

The difference in intention—from a ‘mental health’ point of view by the teachers, combined with a lack of understanding of the pupils’ perspectives, and an unclear sense by the pupils themselves—may have contributed to some pupils overall negative experience of the programme. In particular, this focus on mental health, although not explicit, may have come across to pupils in an implicit way, contributing to their interpretations of needing to block, repress, ignore, ‘forget about’ or ‘shift away from’ negative or bad thoughts, and instead focus on positive thoughts. This may have also contributed to the perceived judgement by some pupils—for example, the intention from the teacher to ‘fix’ or ‘bandaid’ perceived mental health problems, could explain why some pupils, such as Pupil 8 from School 1 or Pupil 3 from School 2, perceived the course to be invalidating and judgemental. It could also contribute to some pupils having an increased rate of negative thoughts, as implicit messaging such as avoiding true discussion around emotions such as anger or sadness, or an undercurrent of pressure passed on from teachers to pupils, may trigger or worry particular pupils about particular thoughts they were having, or indeed if they were doing it ‘correctly’ if indeed negative thoughts increased, as in Pupil 11 from School 1.

Pupils were also not observed to ask for help in class—none of the pupils with difficult experiences described in the first section had mentioned them to their teacher in class. This may have been because specific difficulties were not mentioned to them as possible outcomes, and nor were they observed to be explicitly encouraged to come forward and share these difficulties. The way that teachers conceptualised and managed difficulties may have impacted on the phenomenological experience of pupils.

The teacher from School 3, who was a very experienced .b teacher, was clearly aware that ‘people definitely find it difficult’, and was able to give examples from his prior teaching experience about how he managed difficulties. He was able to acknowledge that pupils sometimes struggled with activities like concentrating on breathing, clapping hands, and having difficulties when their minds start to settle more. Over time, this teacher had developed a number of techniques to help manage these difficulties. For example, he explicitly asked pupils after the exercises if anyone felt worse, so that he could ask them to stay behind and talk to them. He was also aware of what may be happening in each pupils life—if he was concerned about a particular pupil, he would talk to them beforehand, keep a close eye on them during the lesson, and then ‘check in’ with them after the lesson. He also mentioned that pupils crying is not uncommon and usually requires having a chat after the lesson to help address the difficulties—and he ‘is sure to give a proviso that pupils did not have to practice if they feel uncomfortable’.

This differed from the teachers from School 1 and 2, who had much less experience, despite also being rated as competent teachers. The teacher from school 1 only had one example of how she managed difficulty, which was when a pupil instantly opened their eyes during a ‘looking at difficulty’ session, to which the teacher gave a concerned look in their direction, as if to say ‘are you OK?’ She also described 10 min chats that she would have with pupils in the corridor of the school about how they were going with .b, but that these were opportunistic in nature and not specifically about difficulties. This teacher also acknowledged that pupils didn’t come and tell her difficulties because of a lack of time available:

I don’t have a .b moment now to come and talk to you about it. We do miss a lot of stuff. Because you’re teaching them and it’s in, out, in, out, they don’t have the chance to say ‘I find something at home really hard’… I think you miss some of that as a teacher. [TI, School 1]

This could also be a reflection of the time pressures that teachers in many state schools commonly operate in.

The teachers from School 2 said they were getting better at managing difficulties—for example, when a pupil came to them after a class to discuss difficulties, rather than getting caught up in solving the problem, they were able to reflectively listen. They also gave the example of Pupil 7, who had dropped out of the class due to safeguarding concerns and things happening at home, who they did not pressure to return. The teachers from School 1 and 2 seemed to be lacking in their knowledge of potential difficulties and how to manage them compared to the teacher from School 3, and this is possibly due to the degree of experience or the degree of training, or indeed a lack of knowledge or awareness in general about difficulties and challenges in meditation informed curricula and the .b programme more specifically.

The way in which content was delivered during a lesson also had the potential to impact upon how pupils experienced the course. Firstly, lessons were frequently rushed. Rushing the lesson may have had implications for the quality, fidelity and accuracy of the content as transmitted to pupils, as well as allowing less time to explore experience and correct mistakes in conceptualisations. This was highlighted by Teacher 2b from School 2:

Just feeling like it was all quite rushed sometimes, and maybe… I don’t feel like… there was a few times when I just felt like ‘I’m not explaining this in the way that I want to because I know that I need to get onto the practice in two minutes, because we have to get out of here in five. [TI, School 2]

Teachers also commonly skipped and changed the curriculum content of their own volition, often due to a lack of time available, as highlighted by Teacher 2b from School 2:

When we had an hour and 45 minutes, I would do the entire booklet, and you have so much time to talk. Whereas this, you had to go back and just look at the pages with the little man in the corner, and that was the only ones you could do, and then you were very aware that actually there were really important things in between that, that were quite crucial to the explanation, but you didn’t have time to do that, you only had time to do the bits that it says you have to do, and if we taught a normal lesson like than then the normal lesson might not make very much sense because you’re kind of cutting out the development part I suppose. [TI, School 2]

Teacher confidence in the material they were teaching may also have affected pupil experience, for example through being able to discuss, clarify and explore material. Teacher 2b from School 2 gave the following example:

Oh, the first time we did it, I mean, it was terrifying, because it’s really hard, and even now I still hold the booklets, because there are still things that… it’s because we’re not doing it often enough I suppose, that you still have to kind of read bits off, like quotes and things like that, which are really important and I want to make sure that you get it right. [TI, School 2]

Subtle variations in how content was delivered between schools may also have affected pupils interpretations. For example, a teaching point from a lesson in School 1 was explained as ‘Our minds always go to what’s wrong and catastrophizing’ where in School 2 the same teaching point was worded as ‘Brains can often think of the negative’. It is possible that, in School 1, suggesting that minds *always* go to negatives may have reinforced to pupils that there is something wrong with their own minds, and therefore they require ‘fixing’, perhaps leading to an increased rate of, or more negative interpretation of, difficult thoughts through indirect suggestion.

The relatively less experienced teachers from School 1 and 2 also described feeling a significant shift in their role identity as a teacher during the programme. Teacher 1 described a change in role identity as ‘.b mode vs regular mode.’ Regular mode was noted to consist of challenging the pupils and pushing them to extend their knowledge, whereas .b mode was noted to consist more of listening, reflecting and not ‘pushing’ the students. Teacher 1 also found it difficult to switch between these two modes as she changed from one lesson type to another. Teacher 1 usually lead practices with her eyes open, standing in front of the class. She didn’t seem to leave the ‘teacher’ role as much as teachers from School 2, who often lead practices sitting on a chair in front of the pupils—sitting at the pupils level—and who often had either their eyes closed or their head downcast in a position of vulnerability and reduced power. This could have contributed to the frequent giggling, laughing and inattention demonstrated by the pupils in School 2. Teacher 3 seemed to strike a good balance by sitting on a desk—closer to the level of the pupils but not quite leaving the teacher role altogether.

An extension of this change in role identity is the notion of whether the .b teacher was there to correct mistakes in pupil interpretation of the lesson, or whether they were there to simply validate any given pupil experience. An example of this comes from an in-class observation in School 1, where the intended teaching point was to likely cultivate non-judgemental awareness through practising awareness of all thoughts and feeling experienced during practices, including during difficult moments:

Pupils were asked to give an answer to the question ‘A person can’t sleep at night, what advice would you give?’ To which two of the answers were ‘Forget about it’ and ‘Think happy thoughts’. [ON, School 1, week 4]

These answers were not consistent with current understandings of what mindfulness meditation is or should achieve. Yet, these answers were quickly accepted and validated as Teacher 1 quickly moved from pupil to pupil during the lesson. This is not a judgement on Teacher 1, but an example of the tension present between needing to validate any given pupil’s experience and also needing to accurately teach a mindfulness curriculum within the time constraints of a regular school classroom. This can also be an example of where incorrect answers that were not explored adequately may influence the interpretations for other pupils in the class.

An extension of this tension in role identity is the place of discipline in the .b classroom. Teacher 3 spoke of needing to balance the softer experiential side of mindfulness practice with discipline and maintaining control of the class:

You have to know your school sanctions and you have to know when to flip into strict mode. [TI, School 3]

Teacher 2b also noted a balance between ‘being authoritarian’ and ‘passing on knowledge’:

As a regular teacher I’m going in there as an authoritarian as well as passing on knowledge, whereas with the mindfulness I don’t feel like I’m going in there as any form of authoritarian at all. Maybe you’re going at it from more of a caring point of view, rather than a teacher [where it’s] much more about control… controlling what’s going on in the class. It seems a bit of a contradiction to be teaching mindfulness while at the same time losing your temper…you were really aware of the fact that that was not the right environment to do that, but at the same time that was really hard. [TI, School 2]

This teacher seemed to be highlighting the difficulty in balancing non-judgemental validation of pupil experience with needing to occasionally keep a class manageable from a behavioural perspective, and how these may often be in conflict.

Mindfulness teaching was also noted to need an awareness of one’s own internal state at the same time as teaching the course content. This was highlighted by Teacher 2a:

I find as well the difference between the two, because you’re teaching mindfulness you’re very conscious of yourself about practicing your 50/50 attention… you’re aware of when you’re becoming stressed. [TI, School 2]

Teacher 2b also noted a difference in role between teaching a compulsory mindfulness class, compared with a class that was voluntary:

We were fighting them… having to sell it to them. They didn’t really want to do it. They didn’t take it seriously. It was very difficult to know how to be a mindfulness teacher and a teacher… it was really awkward because you didn’t want to shout, you didn’t want to have a go at them because that’s not the environment we’re in, but at the same time, you had to eventually. [TI, School 2]

Practices were also observed to occasionally be used as disciplinary tools by Teacher 1, as this in-class observation demonstrates:

Sit up, no talking, everyone in their bubbles, it seems like everyone has forgot that recently. [ON, School 1, week 9]

The tension in role identity for .b teachers seemed to have implications for the teacher’s responsibility to correct mistakes and provide adequate disciplining, both of which seemed to contribute to overall class climate and pupil experience of the .b programme. Also important was whether or not pupils participated in the course on a voluntary, opt-in basis, or as part of normal school provision (an involuntary basis). The use of mindfulness—through the language of .b—as a tool for discipline also seemingly contradicts the definition of mindfulness as the cultivation of non-judgemental awareness, or to simply be present as experience, and had the potential to confuse pupils further with regards to the desired intention of performing the practices.

### The Curriculum

Certain aspects of the curriculum had the potential to be related to non-positive pupil experiences, both directly and through potentially causing contradictions and mixed messaging with other content, which in turn may have influenced misinterpretation and added to the difficulty and challenge experienced by pupils.

The first factor that was highlighted by Teacher 1 was the amount of experiential practice compared to the amount of other content she had to cover, which relates to the overall programme structure:

I think sometimes we go into a bit too much detail, that maybe we could do the practices and either spread it out or just change it somehow. Change it somehow to get rid of some of that. A little bit less content in general, more practical stuff. [TI, School 1]

She said this was a reflection of pupils asking her for more practise of techniques, as they found them useful ‘when they notice things are going out of kilter within themselves’. However, she reflected that she had to teach what was structured in each lesson and didn’t feel comfortable deviating too much from that. She added that repeating the same practices over different weeks, to improve a sense of mastery, familiarity and skill, may also have been more useful:

And even if you do FOFBOC [the ‘feet on floor bums on chair’ exercise] more than once, beditation more than once, that would be I think more useful than perhaps as much detail as we do. [TI, School 1]

A similar idea was highlighted by pupils from School 2:

Pupil 1: Because even if we did like the same thing on both the days that we did it, I feel like… because then you get more practice and then you get more time on it as well. And like recapping the stuff you’ve done.

Pupil 2, 3: Yeah. [FG2, School 2, Pupils 1, 2, 3, all female]

Teacher 1 also highlighted that pupils in her school were unable to access the internet because they did not have it at home, which impacted on their ability to practice at home. This may be a significant issue in areas with higher levels of deprivation, which may exacerbate inequalities for the most vulnerable.

Another factor highlighted by some pupils was that the length of the course was too short, as indicated by the following pupil from School 3:

I think my thoughts are very scattered. I think if we had a lot longer to do this course then it would obviously help more. I think it does help, but I think it’s more time, more time would obviously help. [FG, School 3, Pupil 20, male]

Teacher 1 and 2b also highlighted how they thought some lessons were more important than others—for example, Teacher 1 said that lesson 7, about learning to watch thought traffic, was a ‘cornerstone’ lesson, and should have been at the start of the course. If this lesson was at the start, it may have helped to make the overall purpose of the curriculum clearer, since the emphasis throughout the course may then have been more clearly on being able to develop identification and cultivation of non-judgemental awareness.

It is also possible that certain content in the curriculum may have made pupils more likely to interpret a sense of needing to block certain thoughts, or to focus more on positive thoughts. For instance, the lesson on flow may have introduced confusion for pupils as it could have been perceived to be about finding a performance (positive) mindset; for example, when pupils in School 1 were asked during a lesson what ‘flow’ meant, they responded with answers like ‘to not care about anything else’ and ‘when your performance is better’. There are a number of other examples of mixed messaging, which again had the potential to reinforce to pupils that mindfulness is about thinking positively and ignoring, blocking or invalidating ‘bad’ thoughts. For example, the content from lesson 9, observed in School 1, had an animation play about focussing on positives and not negatives, and that mindfulness meant ‘being present with your heart’, which meant ‘noticing the good things in every moment’.

This way of defining mindfulness may have been confusing for some pupils when compared to earlier lessons, and may have led to confusion concerning how the ideas of gratefulness, positivity and non-judgement may interact, especially when mindfulness was defined in the previous example as being ‘grateful with your heart’. For example, compare this to a description of a practice in lesson 7 given by the teacher from School 3, where the focus was on receiving, observing, and being ‘open’:

One thing that comes and goes is sound. Listen to sounds as they come and go. There is no need to label them or go off in the thinking stream, just receive, a bit like a microphone. Thoughts are the same, watch them as they arise and fall, just like sounds. [ON, School 3, lesson 7]

Consider in addition the following excerpt from an in-class observation from School 2:

(The animation slide continued): ‘Autopilot is about missing the good and magnifying the bad.’ As it spoke, [name] and [name] were playing games with each other’s hands. ‘You can sleep through life on autopilot’ the presentation slide voice was saying. ‘Mindfulness is being alive and noticing it. Connecting with each moment as it happens’. ‘What advice did the slide give?’ asked [Teacher]. ‘To focus on the good things’ [answered a pupil]. [ON, School 2, week 5]

In this paragraph, the concept of autopilot is introduced as focussing too heavily on ‘bad’ thoughts, feelings and events in life. The presentation then continued that autopilot is instead about being ‘asleep’ through life (in contradiction to the aforementioned focussing too heavily on bad). Mindfulness was then introduced as the counter to being ‘asleep’. The conclusion formed by the pupil was to ‘focus on good things’. This is an example of how the conclusion formed by the pupil was one of focussing on positive things, which may have occurred due to the ambiguous and mixed nature of the teaching.

Similarly, consider the following excerpt from an in-class observation note from School 1, when pupils were shown a video of a Holocaust survivor who had developed a very positive attitude towards life, by saying that she had learnt to look towards good things instead of bad:

[Teacher] then prompted more discussion by asking what [the woman from the presentation] meant by saying ‘I know the bad things, but I look to the good’. Reponses: You only know good things if you know bad. Focus on positives and you can overcome negatives. Push bad things aside. Know that they’re there, but not in your mind. If you focus on the negative, you will never see the positive. An animation now played about focussing on positives and not negatives. [ON, School 1, week 9]

Even though the Holocaust survivor had stated that she recognised bad things but *chose* to focus on the good, pupils here seemed to completely miss the subtlety in this, and interpreted this in many different ways, for example ‘pushing bad things aside’ and ‘overcoming negatives’. One could argue that there was the potential for more ambiguity to be introduced by this exercise than compared to the previous example from School 3, where pupils were encouraged to cultivate the particular type of non-judgemental awareness that mindfulness meditation encourages by being open and to receive all thoughts and phenomena, like a microphone.

## Discussion

This study found that many pupils encountered non-positive experiences while participating in the .b mindfulness programme, which may have been contributed to by multiple complex contextual factors, including the content and structure of the curriculum, the interpretation of the curriculum secondary to the individual pupil’s lived experience, and other wider systemic factors such as the way teachers are trained to mitigate and understand non-positive experiences, and the way the curriculum is implemented by the school.

### The Content of School-Based Mindfulness-Informed Curricula

This present study has highlighted the critical importance of the accuracy and integrity of the content and structure of school-based mindfulness-informed curricula, particularly regarding the importance of making the intention for participating in such programs explicit at both the beginning and throughout all the lessons; having clearly articulated programme aims and outcomes; and having internally coherent teaching points within the curriculum, which build on each other from week to week in a logical and inclusive manner. This is particularly important regarding making cornerstone teaching points such as the identification and cultivation of non-judgemental awareness explicitly clear, and including how this may or may not relate to other similar but potentially different outcomes such as practising gratefulness, ‘distancing’ or decentring from thoughts, or ‘training attention’. The responsibility for this should lie primarily with programme developers, rather than with school teachers or staff.

In this study, it is hard to say with certainty which of the pupil experiences occurred directly as a result of the experiential mindfulness practice, and which may have been in response to other psychoeducation components or teaching elements of the course. This may partly be due to this particular curriculum blending in aspects of positive psychology, gratitude, and performance-based perspectives such as ‘flow’, which were not carefully contextualised to individual pupil lived experience, nor differentiated from the actual individual, experiential practice of mindfulness meditation. This may have set up an a priori expectation throughout the course about what practices should be like before pupils had a chance to explore true mindfulness practice for themselves. The blending of different approaches within the curriculum, coupled with potentially contradictory teaching points and unclear communication of programme aims and expected learning outcomes, may have led to, at best, confusion or ambivalence amongst pupils, or at worst, perceived judgement, invalidation or worsening of negative thoughts or feelings either before, during or after a practice or lesson.

Hawe et al. have highlighted that in complex interventions, such as school-based mental health curricula, the function and process of the intervention (i.e. the hypothesised change process), not the individual components, should be standardised in order to achieve intervention integrity (Hawe, 2004). Authors have also highlighted critical features of SBIs such as having a sound theoretical base, a focus on intended outcomes and explicit guidelines (Hurry, 2021). This study has demonstrated that, at least from the pupils’ perspectives and possibly from the teacher’s perspectives also, there was not a clear ‘change process’ identified regarding how pupils may progress or how they may be expected to change as a result of engaging with the curriculum. If such a change process was made explicit it might allow the form of the curriculum (the individual components) to be tailored to local conditions and perhaps to individual participant contexts. In the absence of a shared understanding of expected processes and mechanisms of change some aspects of the curriculum may have been perceived as ‘quick fix’ measures. Both Duncan et al. and Hawe et al. highlight that when complex interventions are tailored to local conditions (i.e. individual pupil contexts, circumstances and situations—a realist approach) without altering the overall function or process (i.e. the desired outcome), then this could potentially improve intervention effectiveness and integrity (Duncan, 2018; Hawe, 2004). This is akin to understanding the ‘warp and the weft’ of MBIs—what factors within MBIs are structurally universal or definitive of an MBI (the warp) and what components can be tailored or altered to local or indication-specific conditions (the weft) (Crane et al., 2016).

Authors have similarly argued that MBIs must take into careful consideration why, when and how they are adapted (Loucks et al., 2022). Similarly, fidelity, defined as ‘the determination of how well an intervention is implemented in comparison with the original programme design during an efficacy and/or effectiveness study’ has been highlighted as an important factor in the scientific validity of complex interventions such as school-based mental health curricula (O’Donnell, 2008; Sutcliffe et al., 2015). How well the teacher knows the individual pupils and their home life, and how in turn this may alter the way the teacher frames certain parts of the curriculum, and also manages the pupil, is important here. A meta-analysis by Mullen et al. of 500 patient education trials showed that interventions were more likely to be effective if they met criteria fitting with behavioural change theory, such as being tailored to the patient’s individual learning needs or providing feedback about the patient’s progress, along with their training, ability and confidence in dealing with challenging situations (Mullen et al., 1985). Equally, improving sensitivity towards current or past trauma is something which is also increasingly being acknowledged by the wider mindfulness field (Treleaven, 2018).

### How to Best Manage Non-positive Experiences

This study has highlighted that some pupils who encountered non-positive experiences may have been more vulnerable to begin with. This may predispose them to having more difficult thoughts and feelings during course participation, and without adequate support and management, may result in these difficulties persisting (or worsening), leaving the pupils feeling unsupported. Ideally, a balance should be sought between providing a curriculum which is relevant and useful to all, while identifying and supporting those pupils who may be more in need. In order to potentially reduce pupil anxiety and fear about whether a given sensation or experience is good or bad—i.e. whether or not they are doing a practice accurately, or whether or not what they are experiencing is ‘normal’ or expected—it may be helpful for pupils to have a range of possible experiences highlighted to them during or before the course. This might include highlighting potential difficulties that pupils may encounter and how best to manage them, including how best to support themselves and/or inform their teacher or family.

The extent that training prepares teachers to support pupils who experience difficulties also needs to be further investigated, including highlighting issues relevant to any differentiation in the planned method of interpretation and implementation. Programme designers should ensure that the potential for non-positive experiences, including the types of potential non-positive experiences, are understood and clearly communicated to teachers with how best to manage them.

### Limitations

There are a number of limitations with this study. Firstly, the quality measure used in this study, the MBI-TAC-TEACH, has not been validated against any measure of pupil outcomes and so it may not be sensitive to dimensions of teaching that may contribute to or ameliorate difficulty. Specific pupil subgroups, for example neurodivergent young people, may also need further consideration in programme design, perhaps through screening or programme adaptation. There are a number of other factors relating to how a school implements a mindfulness-informed curriculum that may also need to be taken into consideration when considering non-positive pupil outcomes. These include the target class size, age of pupils, time of day the lesson is taught, whether it is optional or voluntary for pupils, the amount of homework (and whether it is enforced or optional), whether individual lessons are omitted or abbreviated, how to accommodate pupils who may be absent for particular lessons, the overall duration of the course (and how frequently lessons occur), whether single classes or whole school year levels participate, and whether classes are continued beyond the initial course delivery.

Furthermore, the researcher would have ideally observed programme training sessions in order to fully understand how teachers are prepared to deliver the programme. This would have enabled differentiation between issues related to programme content and fidelity of programme implementation. Future studies should have sound knowledge of teacher training protocols when investigating school-based mental health curricula and their effects on pupils. Additionally, future research could explore how children respond to meditation at different developmental stages, as this study explored adolescence only. Given the nature of the qualitative approach, the limitations with this research are of course the generalisability of the data, including any implicit or explicit explanatory outcomes which may have been conferred. The authors also acknowledge that many pupils did not report non-positive experiences and rather found the course to be beneficial in some way, or at the very least not harmful.

## Conclusion

Mindfulness-informed school-based curricula show much promise in cultivating school cultures and climates geared towards pupil well-being and mental health. However, this novel field requires further refinement and scientific exploration to make sure that core, fundamental concepts of mindfulness meditation are not lost in the process, and similarly that non-positive pupil experiences are explored, understood and accommodated for in programme design, rollout and implementation.

## Research Questions (Focus Group One)

Double check

1. Assent form signed by pupil and researcher
2. Parental consent
3. Say script
4. Discuss ground rules

Topic guide

- Intention
- General experience
- Round-up

Student Questions

Intention

- Tell us what you’ve been doing in your .b course so far (icebreaker)
  - Prompts (factual):
    - When do you do it
    - How many classes have you done
    - How long do sessions last
    - What do you do in each session
    - What did your teachers tell you were the reasons for doing a .b course in school?
  - Prompts:
    - Did you agree or not agree with that explanation based on your experience?
    - What are your understandings of why you are doing this course?
  - What is mindfulness meditation to you?
  - Prompts:
    - What do you think it means?
    - Why do you think people do it?

General Experiences

What did you (have you) experienced during the course (so far)?

- Can you tell me more about that?
- Any aspect of the course that stood out to you?

Did you notice any particular thoughts, feelings, emotions and body sensations during the *mindfulness exercises* (use student’s own words)?

How did you find doing the meditation exercises at home during the week?

- Did you manage to do any?
- If yes, what helped you do that?
- If no, what got in the way?

Did people at home know you were doing the course? Did you talk about it? What did they think about it?

Round-Up

- Is there anything else about your experiences of the course, that we haven’t discussed, that you would like to mention?
- Is there anything you would say to another student who is about to start a .b course at their school?
- General round-up, tell them how very helpful their reflections will be, thank them for being open and sharing their experiences, go over how the data will be used again so they are sure, check they are still comfortable with that having now gone through the interview, remind them that they can ask at any time if they have questions and remain free to withdraw. Provide them with debriefing information sheet which also has this info and the researcher contact details. Follow-up any safeguarding concerns immediately.

## Research Questions (Focus Group Two)

Double Check:

1. Assent form signed by pupil and researcher
2. Parental consent
3. Say script
4. Discuss ground rules

Topic guide

- General experience
- Explore positive experiences in more detail
- Explore difficult and negative experiences

Student Questions:

General Experiences

- What are your reflections of the course?
  - Prompts
    - What were the highlights/parts of the course that stood out for you?
    - What parts were difficult?
    - What parts didn't you like?
    - Were you able to keep to the home practice?
  - Tell us if you learnt anything about your thoughts or your feelings during this course?
    - Prompts:
    - What sort of thoughts did you notice you were having?
    - What sort of feelings or emotions did you notice you were having?
    - What sort of body sensations did you notice you were having?
    - Were there any experiences you liked?
    - Were there any experiences you didn't like, were difficult or uncomfortable?
  - Positive Experiences
    - You mentioned that during the course you experienced x – neutral to positive experience [x – whatever student’s exact words]—can you tell us more about that?
      - Prompts:
        - What do those experiences mean to you?
        - What did you learn from that experience?
      - Did you experience anything [else] during the course that was interesting, helpful, or positive in some other way (phrase depending on responses to question above)?
      - Challenging Experiences
        - Prompts:
          - You mentioned that during the course you experienced x – neutral to potentially ‘negative’ experience [x – whatever student’s exact words]—can you tell us more about that?
          - What do those experiences mean to you?
          - What did you learn from that experience?
- Did you experience anything [else] during the course that was unusual, challenging or difficult in some other way? (phrase depending on responses to question above)?
- Round—Up
- Is there anything else about your experiences of the course that we haven’t discussed, that you would like to mention?
- Is there anything you would say to another student who is about to start a .b course at their school?

## Research Questions (Individual Interview)

Topic guide

- Context
- Exploring experience more deeply
  o Referencing experiences previously mentioned by pupil, discuss in more detail positive, negative etc.
  p Use notes from pupil’s journal to construct, guide conversation
- Insight
  o Explore any meaning-making processes
- Round-up

Student questions:

Context

- Can you tell me about how you were feeling about life in general before doing this course?
- How were you finding school?

Exploring experiences more deeply

- You mentioned [x experiences] before. Can you tell me more about that?
- In your journal you wrote [x experience]. Can you tell me more about that?

Insight

- Did you learn anything about yourself during or because of the course?
- Did you have any experiences during the course that made you think about things in a different or new way—or 'shifted' the way you think about things?
- Do you feel like you—or your relationship with others—has changed in any way because of this course?

Round-up • Is there anything else about your experiences of the course that we haven’t discussed, that you would like to mention?

Round-up, tell them how very helpful their reflections will be, thank them for being open and sharing their experiences, go over how the data will be used again so they are sure, check they are still comfortable with that having now gone through the interview, remind them that they can ask at any time if they have questions and remain free to withdraw. Provide them with debriefing information sheet which also has this info and the researcher contact details. Follow-up any safeguarding concerns immediately.

## Research Questions (Teacher Interview)

Ensure that consent form is completed and signed and dated (all sections initialled) by both teacher and researcher. Go over issues around confidentiality and its limits, how the data will be used, right to withdraw etc.- we can produce a script for this so info is consistent and everything is covered.

Topic guide

- Intention
- General experience of students
- Positive experiences
- Difficult experiences & response
- Reflections of course from teacher perspective
- Round-Up

Teacher Questions:

Intention

- What were the main reasons you / your school decided to introduce the .b course for students?
- Did you have any particular hopes or aims for the course?

General experience of students

- How do you think the students found the course in general?
- What did you think their expectations were?

Positive experiences

- Can you describe any particular positive experiences that you think students had?

Difficult experiences

- Can you describe any particular difficult experiences that you think students had?
- Why do you think students’ experiences differed?
- Can you describe how the young people responded to any difficulties they had with the course?
- Did you need to do anything extra to support any of the students through the course?

Reflections of course

- Do you have any advice or reflections that you would share with other teachers who are thinking of introducing the course for the first time?

Round-up

- Is there anything else about your experiences of the course that we haven’t discussed, that you would like to mention?
- General round-up, tell them how very helpful their reflections will be, thank them for being open and sharing their experiences, go over how the data will be used again so they are sure, check they are still comfortable with that having now gone through the interview, remind them that they can ask at any time if they have questions and remain free to withdraw. Provide them with debriefing information sheet which also has this info and the researcher contact details. Follow-up any safeguarding concerns immediately.

## Additional Data: Non-positive Experiences

Difficult thoughts did not only occur in the context of misunderstandings of practice intentions, although this was a prominent observation. An older pupil, from School 2, also described becoming more aware of negative thoughts in a way that led to interference with her daily activities, stating: [When doing the exercises] I just thought about every other bad thing in my life. Like when I do it I’m just like… When I do it, because I end up not thinking about everything else, I’m thinking about… like all the bad thoughts come into my head instead of what’s going on around me. [FG2, School 2, Pupil 2, female]

Likewise three pupils from School 3, class 1 (all male) also described a worsening of difficult thoughts as a result of doing practices: Pupil 1: With the snowballing exercise, I [began] realising what things I would have let spiral out of control. So I felt those. Going through it, I was thinking about it again. Pupil 2: Yeah, there were trigger words brought into my head; ‘This isn’t the thing I want to be thinking about.’ Pupil 3 Sometimes as well I just think of a bad memory – it may be something that triggers it – sometimes I just think of a bad memory. I try to block it out but it’s quite hard to do that. I just sort of relive it a bit. It stays for probably half an hour. Until I get distracted by something else it stays there. I don’t like thinking back to some bad things. Pupil 2 – That also happened for me. [FG, School 3, Pupil 1, 2 and 3, all male]

In a similar way to the previously listed pupils, these pupils described triggering of negative thoughts, which had the tendency to linger. The pupils from School 3 described a bad memory or negative thought being triggered, attempting to block it out, reliving it, and then having it stay with him for a while. This potential for exacerbations of negative thinking was acknowledged by the teacher of School 3: Relatively few people feel worse in inverted commas, but some do, and that’s when you have to sort of go, ‘Okay’, and that’s when you probably ask them to stay behind and have a chat about what was going on… It might be that they’ve had a memory about something. [TI, School 3]

Pupil 22 from School 1 described the following situation: Sometimes, I could be going to say something and then obviously forget what I’m going to say, and then do a .b, which will then forget what I’m doing and just make it worse, and then, if I don’t do a .b, it just doesn’t get worse, but makes me want to do another .b, so just cycle, repeat, and I can’t help doing a .b, which just makes it worse. [FG 2, School 1, Pupil 22, male]

In another example, a pupil described feeling frustrated that some practices seemed to make things worse – she described situations where she was in a shopping mall or walking down a corridor at school, felt anxiety in that moment, and subsequently did a practice. However, rather than helping her, it seemed to make the anxiety symptoms worse: I just feel like everybody is watching me and everything is very loud, and I can hear everyone. I feel like everybody is talking about me. So, I just do that (a practice) and then it just makes me feel it more, it just makes me want to leave. [II, School 2, Pupil 3, female]

Pupil 7 from School 2 (female), in response to the question in the diary ‘Did you have any experiences that were difficult and challenging?’ wrote: Not really just cried. [DE, Pupil 7, School 2, week 2]

And: Not really, just understanding the feelings. [DE, Pupil 7, School 2, week 4]

Pupil 7 dropped out of the course after week 4 and was not able to be followed up interviews. Teachers from School 2 described Pupil 7 having the following difficulty: [She] struggled with the practices. Yes, she did. She struggled with practices, you know, she’s not comfortable closing her eyes, she didn’t do a Beditation on the floor, she was uncomfortable with that. [TI, School 2]

Some practices seemed to also be difficult for pupils because of the focus on sensitive or vulnerable parts of the body. The following excerpt from School 2’s classroom notes is a good example of this: I noticed [Teacher’s] hand was around her neck as she was reading this to the pupils, then it would shift to her mouth. Some of the pupils were giggling. ‘Notice what it is like from the inside’ [said Teacher]. Breathing – the feeling in the chest and belly was noticed. Pupils were looking around the room now, eyes visibly opened, looking behind them. Some were flicking through pieces of paper on the table. Some were laughing. [ON, School 2, week 3]
